# **Dietary sodium restriction alters muscle lipidomics that relates to insulin resistance in mice**

**DOI:** 10.1016/j.jbc.2021.100344

**Published:** 2021-01-29

**Authors:** Paula Ramos Pinto, Marcos Y. Yoshinaga, Vanessa Del Bianco, Ana Paula Bochi, Guilherme S. Ferreira, Isabella F.D. Pinto, Letícia G. Rodrigues, Edna R. Nakandakare, Maristela M. Okamoto, Ubiratan F. Machado, Sayuri Miyamoto, Sergio Catanozi, Marisa Passarelli

**Affiliations:** 1 Laboratório de Lípides (LIM-10), Hospital das Clínicas (HCFMUSP) da Faculdade de Medicina da Universidade de São Paulo, São Paulo, Brazil; 2 Departamento de Bioquímica, Instituto de Química, Universidade de São Paulo, São Paulo, Brazil; 3 Department of Physiology and Biophysics, Institute of Biomedical Sciences, University of São Paulo, São Paulo, Brazil; 4 Programa de Pós-Graduação em Medicina, Universidade Nove de Julho, São Paulo, Brazil

**Keywords:** low-sodium diet, insulin resistance, lipid metabolism, lipidomics, skeletal muscle, AC, acylcarnitine, AMPK, AMP-activated protein kinase, BM, body mass, BP, blood pressure, CL, cardiolipin, CPT-1, carnitine palmitoyltransferase 1, CVD, cardiovascular disease, FFA, free fatty acid, FPLC, fast protein liquid chromatography, IGF-1, insulin-like growth factor-1, IR, insulin resistance, IRS-1, insulin receptor substrate 1, JNK, c-Jun N-terminal kinase, kITT, insulin tolerance test, LDLR KO, LDL receptor knockout mice, LS, low-sodium, NS, normal-sodium, PC, phosphatidylcholine, PE, phosphatidylethanolamine, PI, phosphatidylinositol, pPE, plasmenyl phosphatidylethanolamine, RAAS, renin-angiotensin-aldosterone system, ROS, reactive oxygen species, SM, sphingomyelin, TC, total cholesterol, TG, triacylglycerol

## Abstract

A low-sodium (LS) diet has been shown to reduce blood pressure (BP) and the incidence of cardiovascular diseases. However, severe dietary sodium restriction promotes insulin resistance (IR) and dyslipidemia in animal models and humans. Thus, further clarification of the long-term consequences of LS is needed. Here, we investigated the effects of chronic LS on gastrocnemius gene and protein expression and lipidomics and its association with IR and plasma lipids in LDL receptor knockout mice. Three-month-old male mice were fed a normal sodium diet (NS; 0.5% Na; n = 12–19) or LS (0.06% Na; n = 14–20) over 90 days. Body mass (BM), BP, plasma total cholesterol, triacylglycerol (TG), glucose, hematocrit, and IR were evaluated. LS increased BM (9%), plasma TG (51%), blood glucose (19%), and IR (46%) when compared with the NS. RT-qPCR analysis revealed that genes involved in lipid uptake and oxidation were increased by the LS: Fabp3 (106%), Prkaa1 (46%), and Cpt1 (74%). Genes and proteins (assessed by Western blotting) involved in insulin signaling were not changed by the LS. Similarly, lipid species classically involved in muscle IR, such as diacylglycerols and ceramides detected by ultra-high-performance liquid chromatography coupled to mass spectrometry, were also unchanged by LS. Species of phosphatidylcholines (68%), phosphatidylinositol (90%), and free fatty acids (59%) increased while cardiolipins (41%) and acylcarnitines (9%) decreased in gastrocnemius in response to LS and were associated with glucose disposal rate. Together these results suggest that chronic LS alters glycerophospholipid and fatty acids species in gastrocnemius that may contribute to glucose and lipid homeostasis derangements in mice.

Dietary sodium increases blood pressure (BP) and cardiovascular diseases (CVDs), especially stroke, myocardium infarction, cardiac hypertrophy, and vascular remodeling that are all related to elevated cardiovascular mortality in general population and hypertensive individuals (1, 2, 3, 4). Although sodium restriction has favorable effects in reducing BP and associated comorbidities, severe sodium restriction in the diet relates to adverse metabolic effects (5, 6). They include the activation of the renin-angiotensin-aldosterone system (RAAS), sympathetic nervous system, induction of insulin resistance (IR), and disturbances in lipid and lipoprotein metabolism (6). Prada *et al.* (2005) (7) demonstrated in rats that a 10-week low-sodium (LS) diet induced hepatic IR that was evidenced by the impairment in insulin receptor substrate 1 (IRS1) and AKT phosphorylation. In the same animal model, LS impaired the lipoprotein lipase (LPL) activity inducing plasma triacylglycerol (TG) elevation despite normal hepatic TG synthesis (8). In LDL receptor knockout mice (LDLR KO), LS increased lipid infiltration in the aortic arch as compared with normal-sodium (NS) diet, which was related to the enhanced plasma TG, free fatty acids (FFAs), and cholesterol (9, 10). These results indicated a role of severe LS restriction in the induction of IR that ultimately relates to CVD. Skeletal muscle is the main site of IR and glucose uptake mediated by GLUT4 is modulated by lipids. There is a lot of experimental and clinical evidence that plasma levels of TG and FFA correlate with insulin resistance and the development of diabetes mellitus (11, 12, 13). This occurs due to the inter and intramyocellular accumulation of TG eliciting the generation of FFA derivatives, such as ceramides (Cer), diacylglycerols (DG), and fatty acyl-CoA that activate PKC isoforms leading to the insulin receptor substrate 1 (IRS-1) phosphorylation at serine instead of tyrosine residues. An altered profile of phosphorylation is also observed in inflammation that accompanies insulin resistance due to the activation of c-Jun N-terminal kinase (JNK) signaling (14, 15). The compromised insulin signaling leads to compensatory hyperinsulinemia and further to hyperglycemia. A vicious circle is created since insulin resistance further impairs the metabolism of TG-rich lipoproteins by the LPL. In fact, in individuals with diabetes mellitus and in obesity, a higher level of intramyocellular TG is observed in comparison with lean healthy individuals, which inversely correlates with insulin sensitivity and muscle oxidative capacity (11, 12, 16, 17). There are no data regarding the influence of dietary sodium restriction in lipid profile and gene expression of skeletal muscle that could affect IR. This study aimed to analyze the global lipidome and gene expression in the gastrocnemius and their relationship with glucose homeostasis and plasma lipid profile in mice submitted to a severe 3-month LS.

## Results

**Figure 1:**
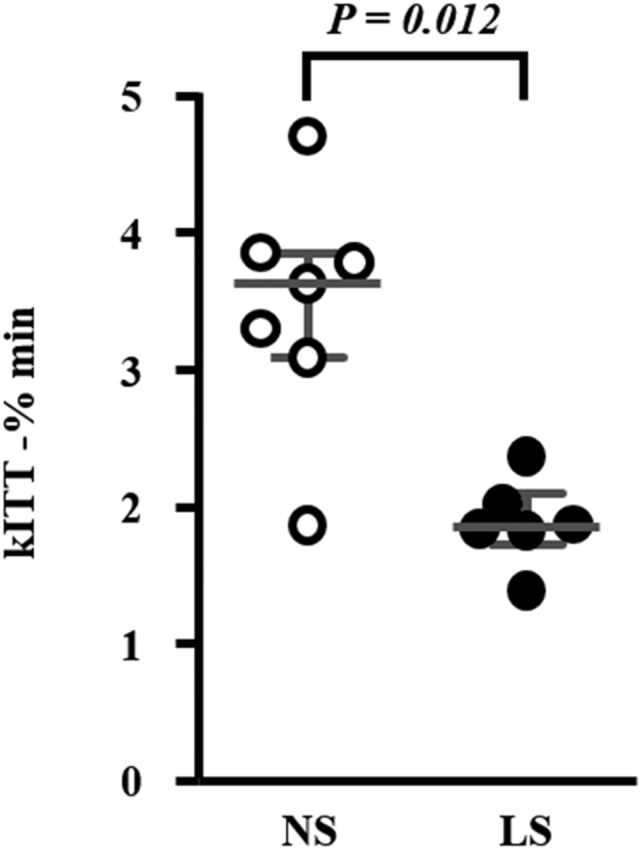
**Insulin tolerance test (kITT) in mice chronically fed either a normal sodium (NS; n = 7) or a low-sodium (LS; n =6) diet.** Twelve-week-old male LDLR KO mice were fed either a NS or a LS diet *ad libitum* for 90 days and kITT was performed after 4-h fasting. The plasma glucose decay rate (kITT) was calculated based on the linear decline in the glucose concentration curve between 10 and 30 min after intraperitoneal injection of insulin (1 U/kg). Values expressed as median (interquartile range) were compared by the Mann–Whitney test.

Body mass (BM), BP, heart rate, biochemical data, gastrocnemius mass, and food intake of animals submitted to NS or LS are depicted in Table 1. In the basal period, animals were similar regarding all these parameters. After 90 days, BM, TG, and fasting glucose were higher in LS as compared with NS animals. The gastrocnemius mass was lower in LS animals as compared with the NS. As expected, 24 h-urinary sodium (UNa) excretion was lower in the LS animals. The insulin tolerance test (kITT) showed a reduced glucose decay rate induced by the LS, confirming IR (Fig. 1).

**Table 1:** Characterization of animals fed either normal (NS) or low-sodium (LS) diet in basal and after 90 days of diet

Groups		NS	LS	*p*
Body mass (g)	Basal	25.0 (22–27)	24.8 (22–32)	0.907
	Final	24.8 (22–27)	26.6 (23–40)	0.005
TC (mg/dl)	Basal	256 (201–284)	257 (200–347)	0.678
	Final	330 (205–690)	345 (149–675)	0.979
TG (mg/dl)	Basal	158 (106–206)	159 (107–236)	0.714
	Final	123 (93–182)	148 (100–439)	0.035
Glucose (mg/dl)	Basal	83 (73–113)	85 (67–122)	0.822
	Final	99 (73–131)	117 (89–156)	0.001
Hematocrit (%)	Basal	50 (38–55)	50 (45–52)	0.891
	Final	49 (35–57)	49 (43–56)	0.815
SBP (mmHg)	Basal	112 (87–118)	111 (98–117)	0.482
	Final	106 (102–122)	102 (95–118)	0.252
DBP (mmHg)	Basal	43 (34–58)	46 (36–58)	0.528
	Final	43 (37–87)	45 (35–65)	1.000
Heart rate (bpm)	Basal	481 (423–616)	474 (439–647)	0.808
	Final	529 (426–623)	532 (431–615)	0.961
Food intake (g/24 h)	Final	2.94 (1.87–4.43)	2.99 (0.72–4.64)	0.535
Gastrocnemius mass (g)	Final	0.30 (0.23–0.34)	0.28 (0.24–0.33)	0.047
UNa (mEq/24 h)	Final	0.17 (0.015–0.412)	0.03 (0.003–0.082)	0.000

Number of animals in NS and LS groups in each analysis: body mass (19,19), total cholesterol (TC; 12,15), triacylglycerol (TG; 12,14), glucose (19,20), hematocrit (18,20), systolic and diastolic blood pressure (SBP, DBP—13,14), heart rate (13,14), food intake (19, 20), gastrocnemius mass (18,19), 24 h-urinary sodium excretion (UNa; 19,20). Values expressed as median (range) were compared by the Mann–Whitney test.

**Figure 2:**
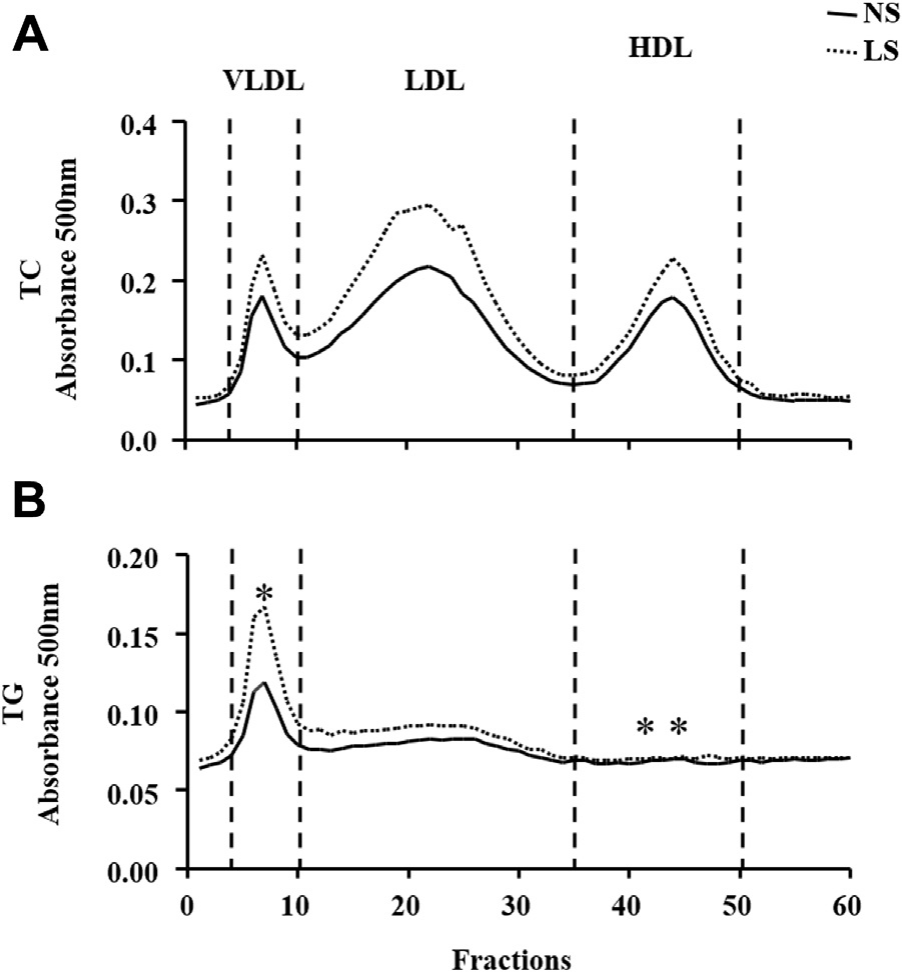
**Plasma lipoprotein profile.** Lipoprotein profile was determined by fast-performance liquid chromatography (FPLC) in plasma drawn from LDLR KO male mice fed either a normal (NS; n = 14) or a low-sodium (LS; n = 15) diet, for 90 days. The percentage of the area under the curve of TC (panel *A*) and TG (panel *B*) in VLDL, LDL, and HDL was used for comparing groups by the Mann–Whitney test.

Plasma lipoproteins profile assessed by FPLC (Fig. 2) showed a similar amount of TC in all lipoprotein subclasses. On the other hand, the percentage of TG in VLDL was higher in LS than NS-fed animals (19 ± 0.7 *versus* 16 ± 0.6, respectively) and decreased in HDL (31 ± 0.8 *versus* 34 ± 0.9, respectively).

**Figure 3:**
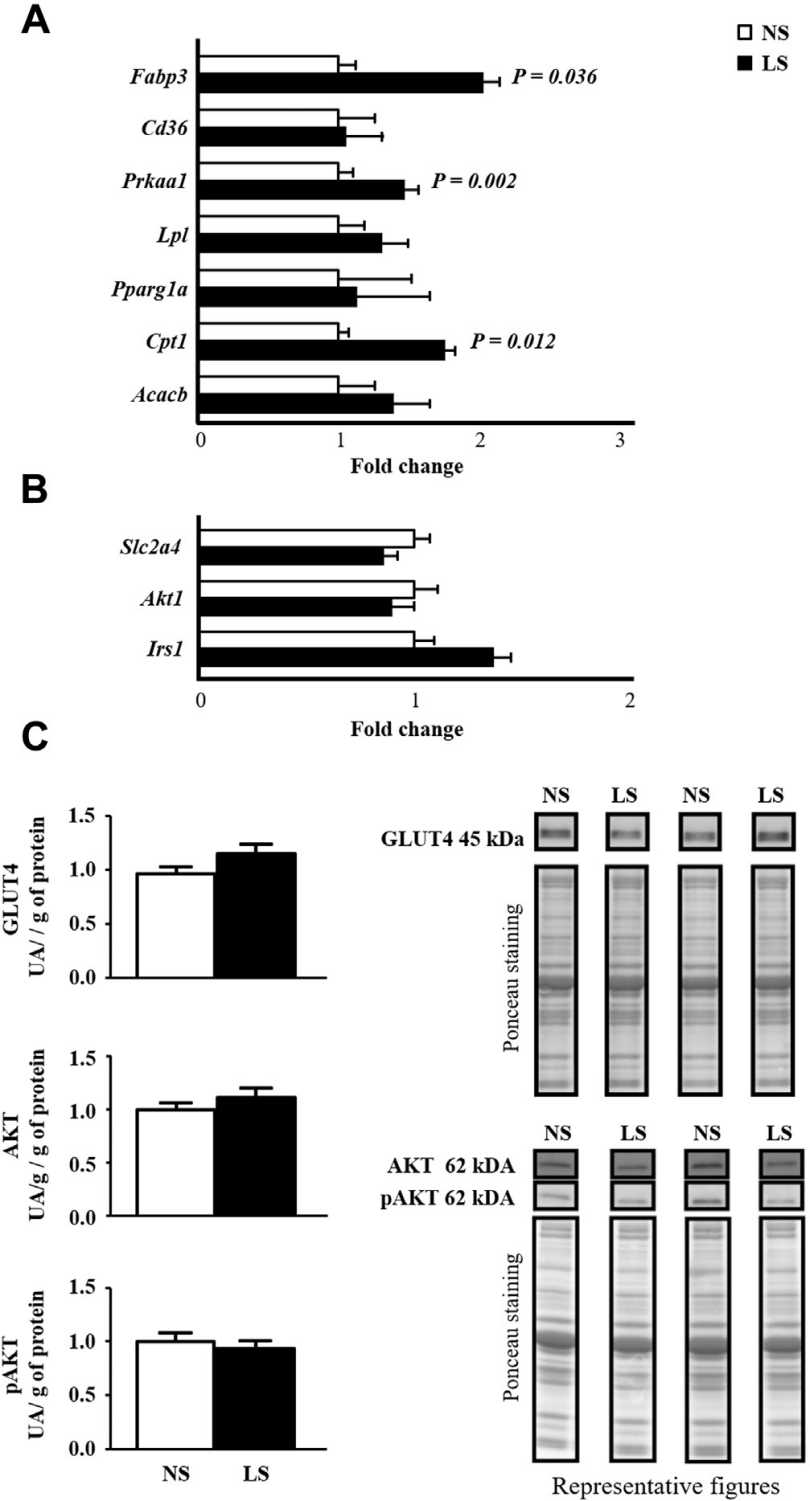
***A*, lipid flux and oxidation genes expression in gastrocnemius.** The expression of genes was determined in gastrocnemius of LDLR KO male mice fed either a normal (NS; n = 5–7) or a low-sodium diet (LS; n = 5–7), for 90 days. Two-hundred nanograms of cDNA was obtained from the left gastrocnemius total mRNA and submitted to RT-qPCR (TaqMan Two Step; TaqMan Gene expression—Applied Biosystems). Gene expression was calculated as 2^−ΔΔCt^. *B2m* was utilized as endogenous control gene. Values expressed as fold change were compared by the Mann–Whitney test. Insulin signaling in the gastrocnemius. *B*, the expression of target genes *Slc2a4*, *Akt1*, and *Irs1* was determined in gastrocnemius of LDLR KO male mice fed either a normal (NS; n = 5–7) or a low-sodium diet (LS; n = 5–7), for 90 days. Two-hundred nanograms of cDNA was obtained from the left gastrocnemius total mRNA and submitted to RT-qPCR (TaqMan Two Step; TaqMan Gene expression—Applied Biosystems). Gene expression was calculated as 2^−ΔΔCt^. *B2m* was utilized as endogenous control gene. *C*, protein content of total AKT, pAKT, and GLUT4 in the right gastrocnemius extracted from LDLR KO male mice fed either a normal (NS; n = 10) or a low-sodium diet (LS; n = 8). Fifty micrograms of total muscle homogenate protein was submitted to electrophoresis into a 10% polyacrylamide gel. After transference to a nitrocellulose membrane, immunoblot was performed by using primary antibodies [total and phosphorylated AKT (1:1000), GLUT4 (1:3000)] and secondary anti-rabbit Ab (1:5000) following chemiluminescence reaction (ECL). Optical density of each band was normalized with Ponceau staining. Results were presented as arbitrary units corrected per gram of tissue. Values expressed as fold change were compared by the Mann–Whitney test.

The expression of *Fabp3* gene (that encodes for an FFA plasma membrane transporter) was greater in LS than NS mice and *Cd36* mRNA was similar between groups (Fig. 3*A*). The expression of *Prkaa1* and *Cpt1* genes that encode for AMPK and CPT1, respectively—was enhanced by the LS. *Slc2a4*, *Irs1*, and *Akt* expressions (that encode for, respectively, GLUT4, IRS-1, and AKT) were similar between groups as well as the protein content of GLUT4, total AKT, and pAKT indicating that insulin signaling was still preserved in the gastrocnemius (Fig. 3, *B* and *C*).

**Figure 4:**
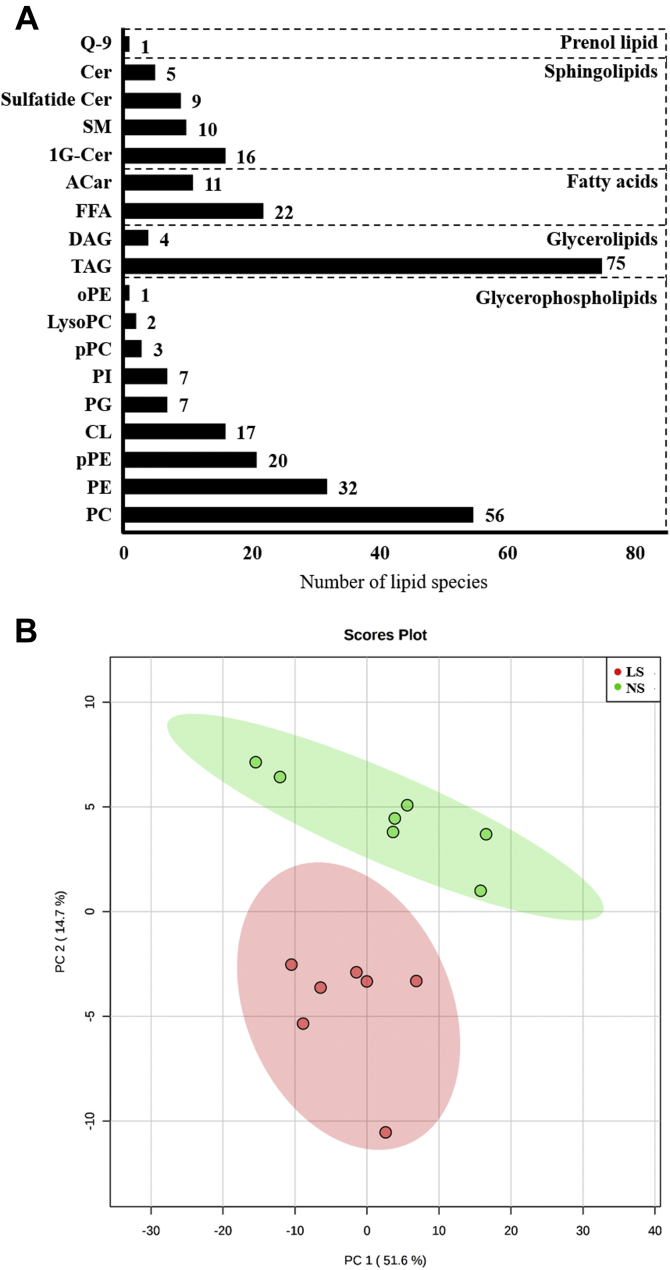
***A*, lipid species in gastrocnemius identified by global lipidomics.** Number of lipid species by category identified in a global lipidomic analysis performed with high-resolution mass spectrometry, ultra-high-performance liquid chromatography coupled to mass spectrometry coupled to ESI-Q-TOF-MS in the lipid extract of gastrocnemius of LDLRKO mice after normal (NS; n = 7) or low-sodium diet (LS; n = 7). 1G-Cer, glycosyl ceramide; AC, acylcarnitine; Cer, ceramide; CL, cardiolipins; DG, diacylglycerol; FFA, free fatty acids; oPE, alkylsulfatylethylethanolamine; PC, phosphatidylcholine; PE, phosphatidylethanolamine; PG, phosphatidylglycerol; PI, phosphatidylinositol; pPC, plasmenyl diacylphosphatidylcholine; pPE, plasmenyl phosphattidylethanolamine; Q-9, coenzyme Q9; SM, sphingomyelin; TG, triacylglycerol. *B*, discriminant analysis by partial least squares (PLS-DA) of lipids in the gastrocnemius. The lipid extract of the gastrocnemius was subjected to a global lipid analysis performed with high-resolution mass spectrometry, ultra-high-performance liquid chromatography coupled to mass spectrometry coupled to ESI-Q-TOF-MS. The PLS-DA shows the separation of lipid species from LDLR KO male mice in NS (*green circles*, n = 7) or LS (*red circles*, n = 7) groups.

**Figure 5:**
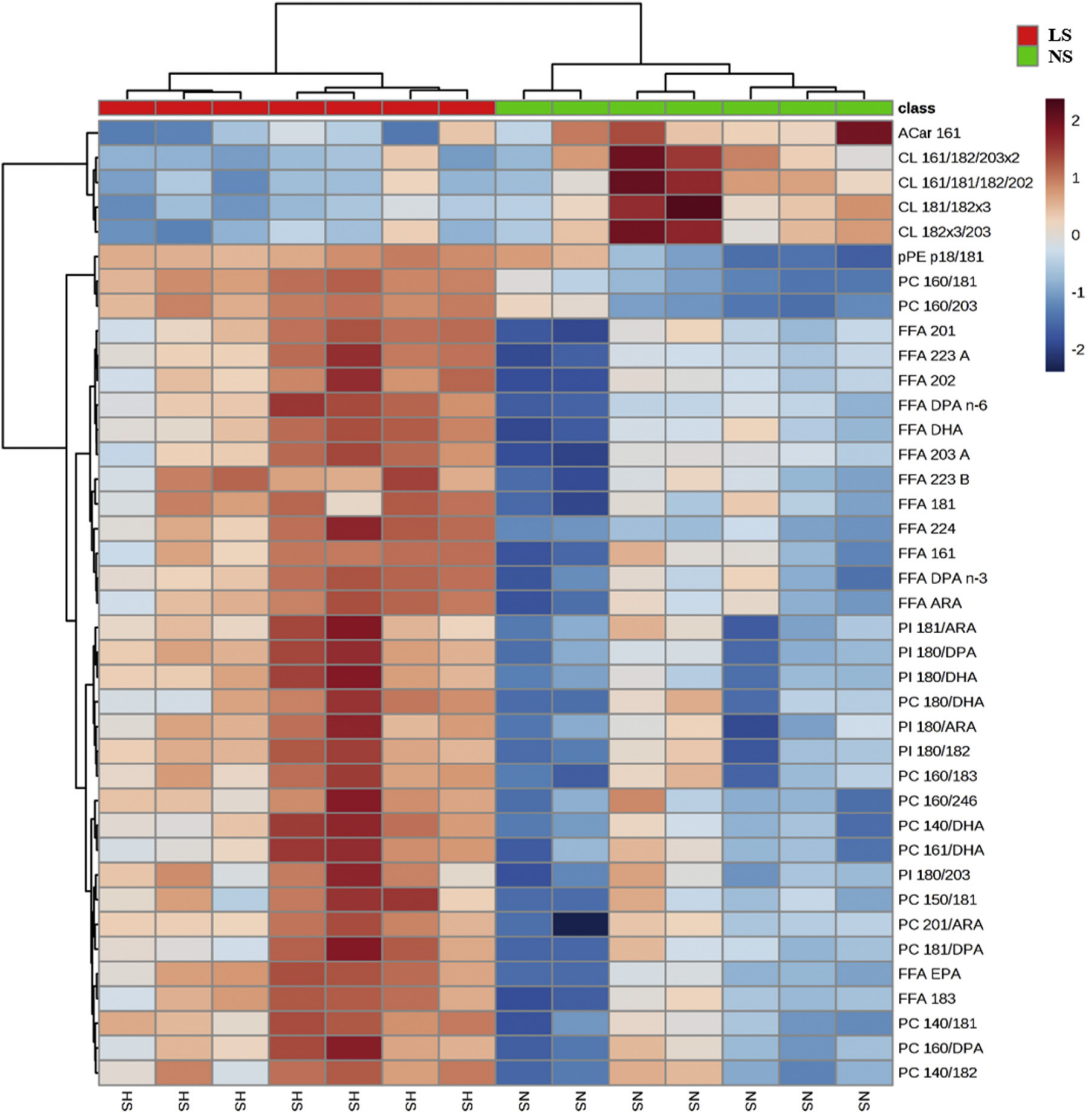
**Heat map of lipid species in gastrocnemius.** The gastrocnemius lipid extract obtained from normal (NS; n = 7) or low-sodium diet (LS; n = 7) fed LDLR KO male mice was subjected to global lipidomic analysis performed with high-resolution mass spectrometry, ultra-high-performance liquid chromatography coupled to mass spectrometry coupled to ESI-Q-TOF-MS. Statistical analysis was performed by using Student's *t*-test, with “false Discovery rate” (FDR) considering *p* ≤ 0.05. AC, acylcarnitine; CL, cardiolipins; FFA, free fatty acids; PC, phosphatidylcholine; PI, phosphatidylinositol; pPE, plasmenyl phosphattidylethanolamine.

Global lipidomics was performed by ultra-high-performance liquid chromatography coupled to mass spectrometry to identify lipid species in the gastrocnemius whole lipid extract. Two hundred and ninety-eight lipid species were identified and distributed in the following categories: glycerolphospholipids (145); glycerolipids (79), sphingolipids (40), FFA (33), and prenol lipid (1) as shown in Figure 4*A*. Partial least squares discriminant analysis (*PLS-DA*) shows segregated lipid species in NS and LS groups (Fig. 4*B*). Besides, the heat map (Fig. 5) provides the hierarchical clustering of those lipid species showing 39 species significatively altered by the LS. The columns show the separation of the red (seven animals fed an LS) and green (seven fed an NS) clusters with the highest levels of acylcarnitine (AC) and cardiolipins (CLs) in animals fed the NS and the highest levels of plasmenyl phosphatidylethanolamine species, pPE, PC, PI, and FFA in the LS group. CL was positively correlated with glucose disposal rate evidenced by the kITT (r = 0.637; *p* = 0.019).

**Figure 6:**
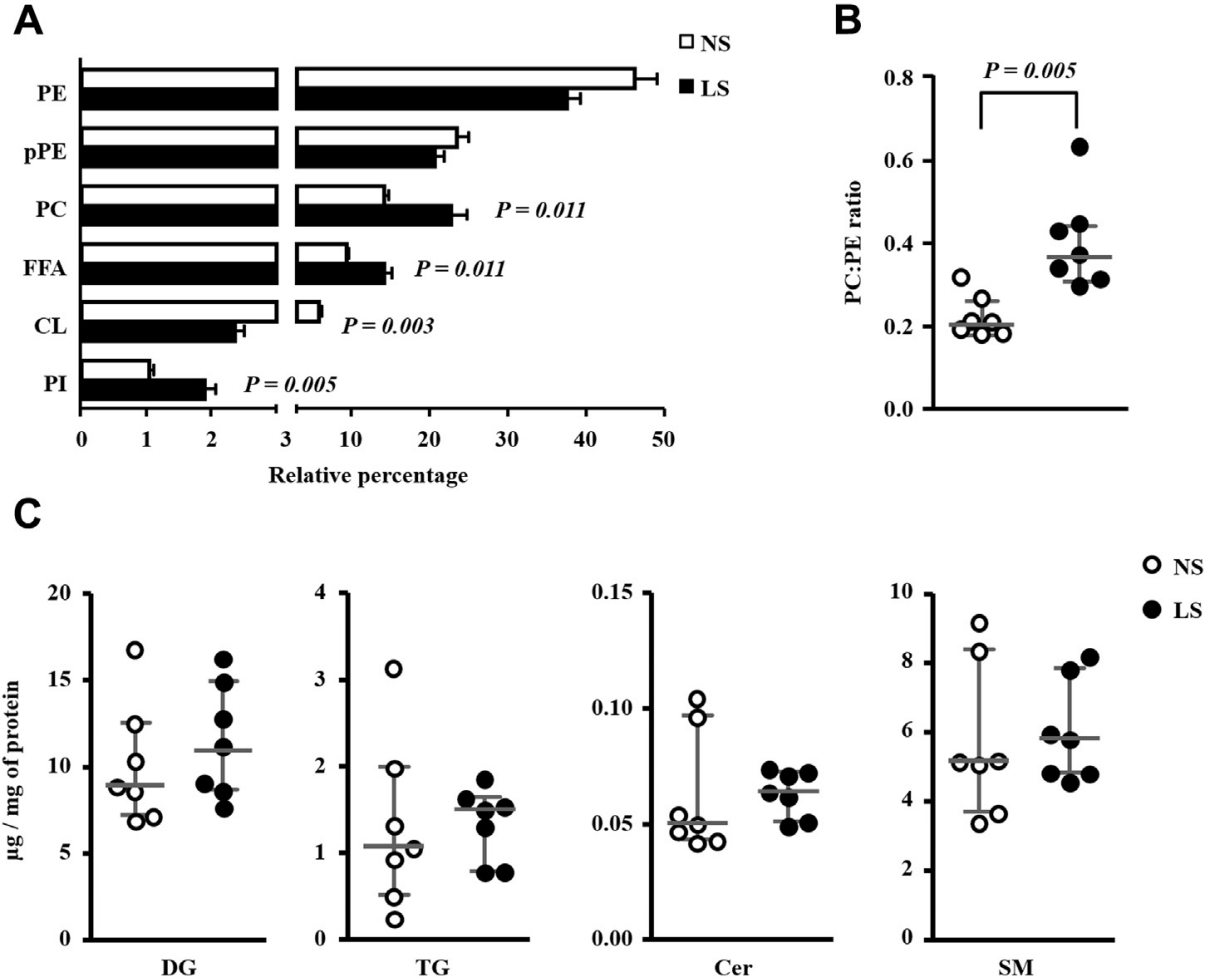
***A*, relative percentage of the six most abundant lipid categories identified in gastrocnemius.** The lipid extract of the gastrocnemius excised from LDLR KO male mice fed either a normal (NS; n = 7) or a low-sodium diet (LS; n =7) was subjected to a global lipidomic analysis performed with high-resolution mass spectrometry,ultra-high-performance liquid chromatography coupled to mass spectrometry coupled to ESI-Q-TOF-MS. Values expressed as average ± standard deviation were compared by the Mann–Whitney test. *B*, PC:PE ratio. CL, cardiolipins; PC, phosphatidylcholine; PE, phosphatidylethanolamine; PI, phosphatidylinositol; pPC, plasmenyl diacylphosphatidylcholine; pPE, plasmenyl phosphattidylethanolamine. Values expressed as median (interquartile range) were compared by the Mann–Whitney test. *C*, total lipid mass of DG, TG, Cer and SM in the gastrocnemius. Values expressed as median (interquartile range) were compared by the Mann–Whitney test. Cer, ceramides; DG, diacylglycerol; SM, sphingomyelin; TG, triacylglycerol.

Global lipidomic analysis revealed that the most abundant species found in the gastrocnemius, in decreasing order, were the subclasses of phosphatidylethanolamine (PE), plasmenyl phosphatidylethanolamine (pPE), phosphatidylcholine (PC), FFA, CL, and phosphatidylinositol (PI), representing, respectively, 42%, 22%, 18%, 12%, 4%, and 1% of the total average lipid mass (Fig. 6*A*). The concentrations of PE and pPE were similar between groups. PC, FFA, and PI concentrations were higher in the LS than NS group. On the other hand, CL concentrations were significantly lower in the LS as compared with NS-fed mice. A positive correlation was found between kITT with CL (r = 0.637; *p* = 0.019) while a negative correlation was observed between kITT and muscle content of FFA (r = −0.604; *p* = 0.029), PC (r = −0.665; *p* = 0.013), and PI (r = −0.659, *p* = 0.014). The concentrations of other lipid species identified were similar in both groups and are depicted in the Supporting Information.

**Figure 7:**
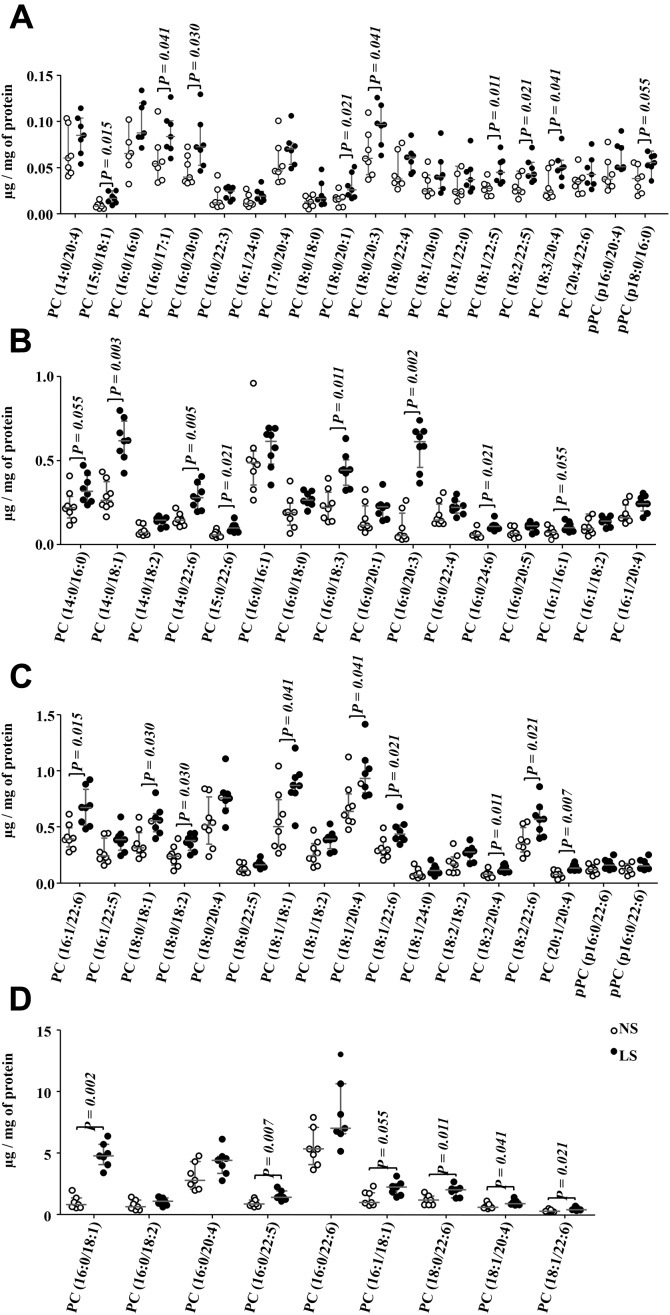
**Phosphatidylcholine (PC) species identified in the gastrocnemius.** The lipid extract of the gastrocnemius excised from LDLR KO male mice fed a normal (NS; n = 7) or low-sodium diet (LS; n = 7) was subjected to a global lipidomic analysis performed with high-resolution mass spectrometry, ultra-high-performance liquid chromatography coupled to mass spectrometry coupled to ESI-Q-TOF-MS. PC species were separated in panels *A*–*D* according to their size, *A*) 0.0 to 0.15, *B*) 0.0 to 1.0, *C*) 0.0 to 1.5 and *D*) 0.0 to 15 μg/mg of protein. Values expressed as median (interquartile range) were compared by the Mann–Whitney test.

**Figure 8:**
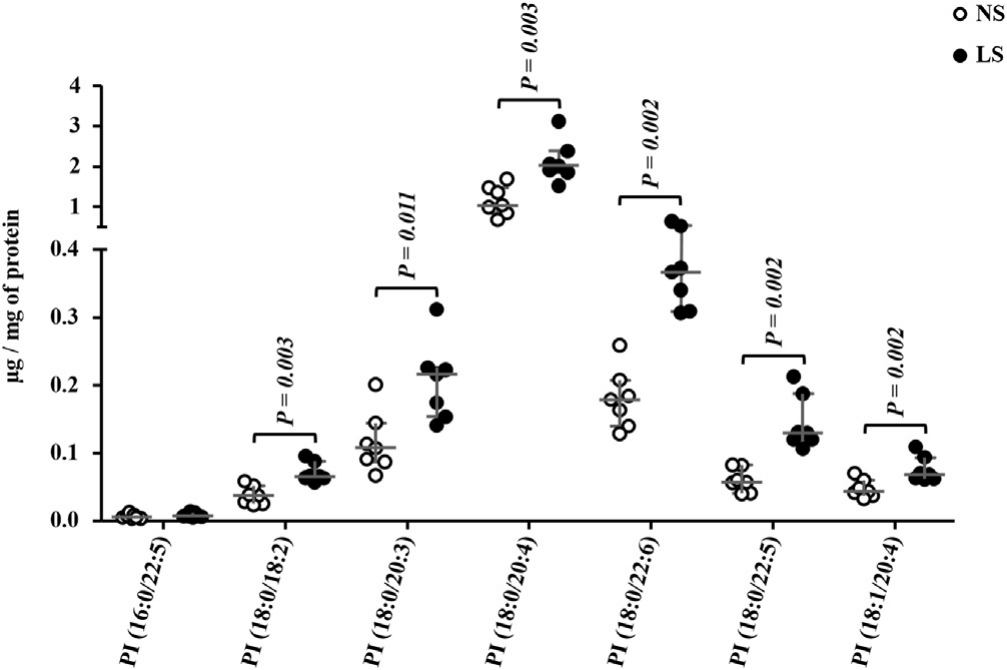
**Phosphatidylinositol (PI) species identified in the gastrocnemius.** The lipid extract of the gastrocnemius excised from LDLR KO male mice fed either a normal (NS; n = 7) or a low-sodium diet (LS; n = 7) was subjected to a global lipidomic analysis performed with high-resolution mass spectrometry, ultra-high-performance liquid chromatography coupled to mass spectrometry coupled to ESI-Q-TOF-MS. Values expressed as median (interquartile range) were compared by the Mann–Whitney test.

The PC:PE ratio was higher in LS as compared with NS-fed animals (Fig. 6*B*). Ceramides (Cer), DG, TG, and sphingomyelin (SM) were similar between groups (Fig. 6*C*). Considering the 59 species of phosphatidylcholine identified in the gastrocnemius, 32 were more abundant in the LS as compared with the NS group and 27 species were similar in both groups (Fig. 7, *A*–*D*). Six species of PI, among the seven identified, were more abundant in the LS than the NS-fed mice (Fig. 8).

**Figure 9:**
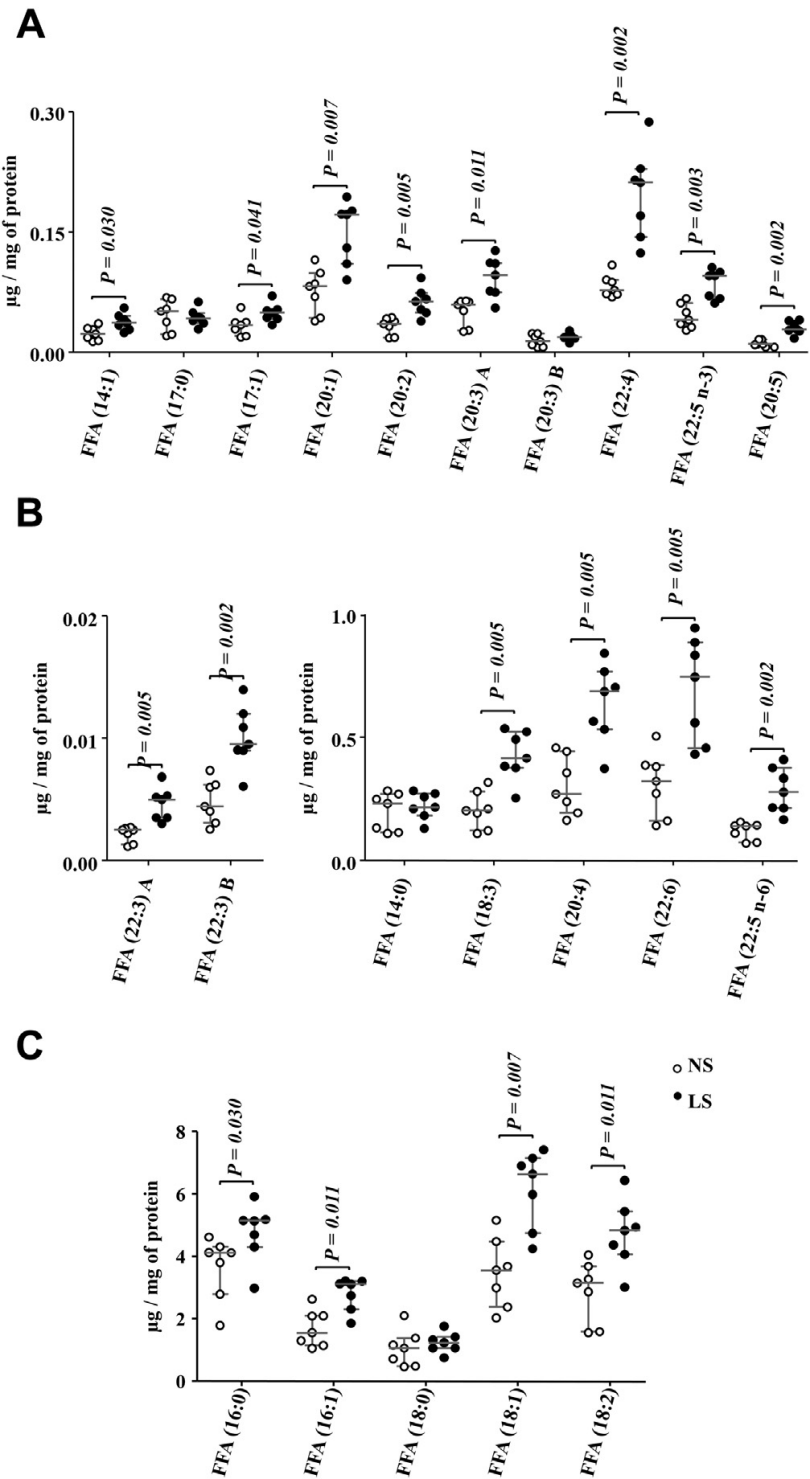
**Free fatty acids (FFA) species identified in the gastrocnemius.** The lipid extract of the gastrocnemius excised from LDLR KO male mice fed either a normal (NS; n = 7) or a low-sodium diet (LS; n = 7) was subjected to a global lipidomic analysis performed with high-resolution mass spectrometry, ultra-high-performance liquid chromatography coupled to mass spectrometry coupled to ESI-Q-TOF-MS. FFA species were separated in panels *A*–*C* according to their size, *A*) 0.0 to 0.3, *B*) 0.0 to 0.02 and 0.0 to 1.0, *C*) 0.0 to 8.0 μg/mg of protein. Values expressed as median (interquartile range) were compared by the Mann–Whitney test.

**Figure 10:**
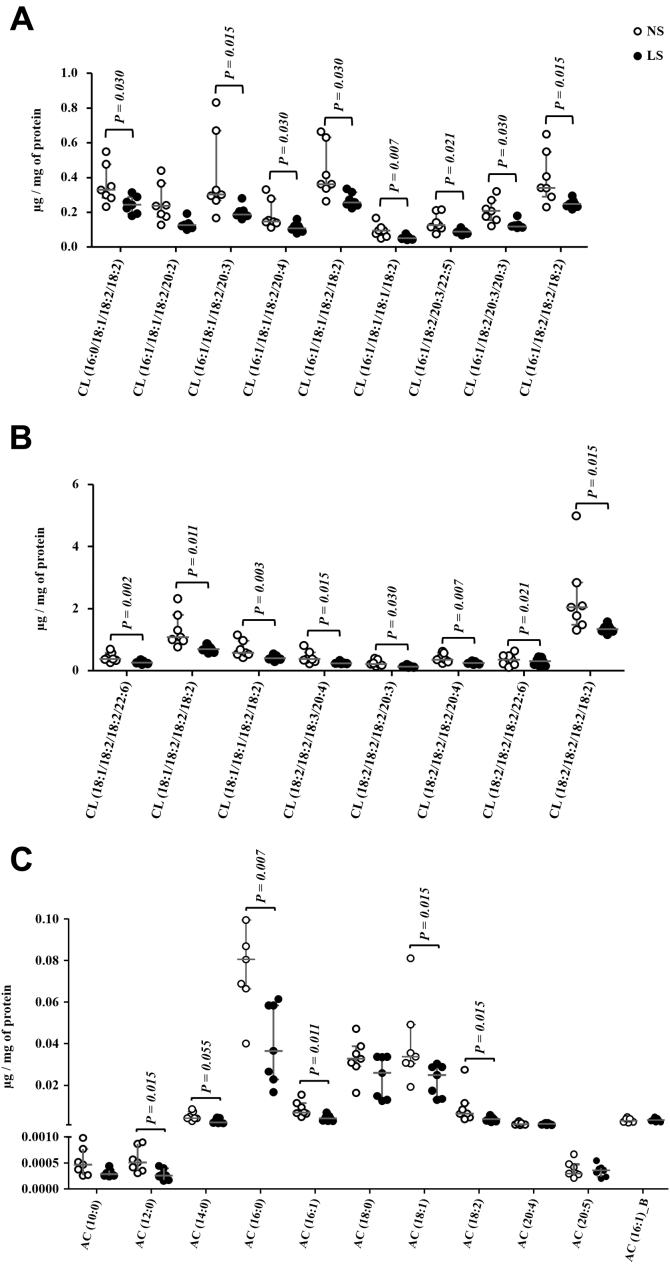
**A and B, Cardiopilin (CL) species identified in the gastrocnemius.** The lipid extract of the gastrocnemius excised from LDLR KO male mice fed either a normal (NS; n = 7) or a low-sodium diet (LS; n = 7) was subjected to a global lipidomic analysis performed with high-resolution mass spectrometry, UHPLC-MS/MS coupled to ESI-Q-TOF-MS. CL species were separated in panels *A* and *B* according to their size, *A*) 0.0 to 1.0 and *B*) 0.0 to 6.0 μg/mg of protein. *C*, acylcarnitines (AC) species identified in the gastrocnemius. The lipid extract of the gastrocnemius excised from LDLR KO male mice fed either a normal (NS; n = 7) or a low-sodium diet (LS; n = 7) was subjected to a global lipidomic analysis performed with high-resolution mass spectrometry, LC-MS/MS coupled to ESI-Q-TOF-MS. Values expressed as median (interquartile range) were compared by the Mann–Whitney test.

Eighty-two percent of the FFA species found in the gastrocnemius were more concentrated in the LS relative to NS-fed mice (Fig. 9, *A*–*C*). Animals on NS presented a greater abundance of CL in 16 species out of the 17 identified in comparison with those on LS (Fig. 10, *A* and *B*). Finally, 11 species of acylcarnitines (AC) were identified in the gastrocnemius; with six being more abundant in animals fed the NS (Fig. 10*D*). Non-modified species of TG, DG, Cer, SM, and PG are shown in the Supporting Information. Although not different between groups, plasma TG was positively correlated with FFA (r = 0.578; *p* = 0.030), PC (r = 0.569; *p* = 0.034), and PI (r = 0.560; *p* = 0.037). Of the 52 species of PE identified in gastrocnemius, concentrations of PE (16:1/18:0) and pPE (p18:0/18:1) were reduced by the LS relative to NS (Supporting Information).

## Discussion

Despite reducing BP, severe dietary sodium restriction is related to the development of IR that alters lipoprotein metabolism favoring lipid infiltration in the arterial wall compartment in mice (7, 9). Skeletal muscle is the major site of glucose disposal contributing to IR that can be affected by lipid species. Then we addressed how severe sodium restriction affects skeletal muscle lipidomics and gene expression that relates to IR. The gastrocnemius was chosen, which has predominantly fast contracting muscle that contains type II (95%) and I (5%) fibers that represents 71% of total muscular mass in murine and reflects glucose homeostasis in the animal’s body (18, 19). Glycolytic fibers develop more insulin resistance according to intracellular lipid accumulation as compared with oxidative fibers (20).

Food intake was similar between groups, although body weight was higher after 90 days in the LS. On the other hand, the gastrocnemius mass was significantly reduced, which may be related, as previously reported, to increased muscle proteolysis induced by the chronic activation of RAAS with concurrent reduction of insulin-like growth factor-1 (IGF-1) and caspase 3 activation (21, 22).

The increment in BM was accompanied by damage in glucose homeostasis. This was evident by the lower rate of glucose disappearance in the kITT of animals fed the LS as compared with those on NS. Similar results were described in Wistar rats fed the LS during 3 months immediately after weaning in which the increased adiposity, body weight, plasma glucose, and the reduction in insulin sensitivity accessed by euglycemic clamp (23, 24, 25) became evident. IR in skeletal muscle as well as in the liver was attributed to diminished IRS-1, IRS-2, and AKT phosphorylation (7), increased phosphorylation of IRS-1 in Ser 307 and JNK-activated signaling pathway.

The IR induced by severe sodium restriction has been ascribed to the chronic activation of the RAAS in both humans and animal models (26, 27, 28). In isolated muscle from Zucker rats or in L6 cells treated with angiotensin II, a reduced AKT phosphorylation and impaired GLUT4 translocation to the plasma membrane as well as diminished activity of the glycogen synthase kinase 3 (29) were observed. Those events were related to the increased NAD(P)H oxidase-mediated generation of reactive oxygen species (ROS) (30, 31).

In the present investigation, dietary sodium restriction increased the expression of *Prkaa1* and *Cpt1*, which encode, respectively, for AMP-activated protein kinase (AMPK) and carnitine palmitoyltransferase 1 (CPT-1). By inhibiting acetyl CoA-carboxylase, AMPK favors FFA beta-oxidation that takes place in the mitochondria after the transference of FFA to its inner leaflet by the CPT-1. These results are in line with the increased expression of *Fabp3* that encodes for a FFA transporter, favoring FFA flux to the muscle. Enhanced amounts of FFA species were found in the gastrocnemius of mice fed the LS and a negative correlation was found between muscle FFA and kITT. Increased concentration of circulating FFA was found in rats and mice on LS as well as in humans, which was related to increased levels of plasma TG (8, 10, 32). In addition, FFA derivatives are related to IR in muscle, by imparing the phosphorylation of tyrosine redisues in IRS-1, disrupting insulin signaling (33, 34). In dyslipidemic mice on LS, plasma FFAs were also related to the enhanced infiltration of lipids in the aortic arch (9, 10).

Although it was evidenced that enhanced muscle FFA and TG concentrations have been positively correlated with PC and PI, the content of TG, DG, Cer, and SM, which are lipid species intimately related to IR (11, 12, 14), was not changed by the dietary sodium restriction. Recently Eum *et al.* (2020) (35) showed that a high-fat diet induced an increase in DG (16:0/18:1, 16:0/18:2, and 18:1/18:1) and Cer (d18:1/24:0 and d18:1/24:1) species in mice soleus. Those lipid species were associated with IR. Three of the species mentioned above were identified in our study (DG 18:1/18:1, Cer d18:1/24:0, and d18:1/24:1); however, none of them underwent modification by the LS. In agreement, no changes were induced by the LS in the expression of genes and protein content of classical insulin signaling molecules and effectors such as IRS-1, total AKT, pAKT, and GLUT4.

The PC:PE ratio modulates FFA oxidation in the mitochondria, an organelle very sensitive to metabolic stressors. PC is very abundant in cells corresponding to 40 to 50% of total PL (36, 37). On the other hand, PE is the second major phospholipid component of the mitochondrial inner leaflet (38). LS diet increased the PC:PE ratio in this investigation that is related to diminished FFA oxidation and reduced insulin signaling in human muscle isolated by biopsy (39). Indeed, the reduction in PC:PE was related to better insulin sensitivity (40, 41). The increment in PC alters mitochondrial function by impairing the activity of complexes I, II, and IV along the mitochondrial electron transport chain (42). According, a negative correlation was found between kITT and muscle PC content.

CLs are tetraacyl phospholipids that are unique in the mitochondria regulating its function through the respiratory chain (43, 44). These molecules are necessary for the proper activity of several complexes of the mitochondrial electron transport chain, electrostatically anchoring cytochrome c to the inner membrane, and maintaining the fluidity and stability of the inner membrane (45). CL loss disorganizes the crest structure of the inner mitochondrial membrane and dissociates complexes, which are essential for the efficiency of the electron transport chain (46) and reduction in CL content in total tissue homogenate is postulated as reflecting a reduction in mitochondria mass (45). In the present investigation, 16 species out of the 17 identified CL were present in lower concentrations in the LS as compared with the NS, reinforcing the possibility of mitochondrial function impairment elicited by severe dietary sodium restriction. Nonetheless, the mitochondrial function was not addressed in the current study and deserves further investigation.

Six species of AC were reduced by the LS although the interpretation is still difficult. In classic models of IR such as diabetes mellitus or obese animals that were fed a high-fat diet, disturbances in mitochondrial metabolism were evidenced, including low rates of FFA oxidation and oxidative stress, as a result of the accumulation of AC in the skeletal muscle (47, 48).

Also, species of PI were more abundant in LS animals, although their implications should be investigated in detail. PI 18:0/20:4 was the most abundant species affected by LS in the gastrocnemius, which has been described by Eum *et al.* (2020) (35) as one of the lipids involved in soleus IR in mice fed with high-fat diet. Besides, 20:4 (arachidonic acid), a lipid mediator of inflammation, was found in glycerophospholipid species including PI, PC, and PE and associated with obesity in Wistar rats (49).

The results show that the IR and enhanced plasma TG levels that were elicited by a severe dietary sodium restriction were related to changes in the gastrocnemius global lipidomics reflected by enhanced amounts of intramuscular PC, PI, and FFA and reduced CL and AC. Association analyses showed a negative correlation between the gastrocnemius content of PC, FFA, PI with kITT while a positive correlation was found between AC, CL. These data reinforce an association of lipid species with the glucose homeostasis in animals fed a LS.

In LS mice it is likely that a reduced AC and increased *Fabp3*, *Prkaa1*, and *Cpt1* could be contributing to a lipid-driven mitochondrial remodeling helping to prevent TG, DG, and Cer elevation although FFAs are still increased probably reflecting mitochondrial impairment. Nevertheless, more studies are necessary in order to better understand the exact impact on the mitochondrial function that may underlie IR according to this different lipid profile that was established in LS-LDLR KO mice. Although classical insulin signaling and lipid species that are known to modulate insulin sensitivity were not changed, data point for a role of severe LS in preconditioning the development of glucose dearrangements in the muscle. These results should be further validated in animal models fed LS for longer periods and humans to provide new insights to understanding the adverse effects of the intense sodium restriction that contributes to derangements in glucose and lipid homeostasis.

In conclusion, severe dietary sodium restriction increased body weight, IR, and plasma glucose and TG in LDLR KO mice. In the gastrocnemius, LS increased the expression of *Fabp3, Prkaa1,* and *Cpt1* and the content of FFA, PC, and PI, reducing CL and AC. Lipidomic changes may contribute to IR in those LS-fed animals.

## Experimental procedures

This study was approved by the Animal Care and Research Advisory Committee of the Faculdade de Medicina da Univesidade de Sao Paulo (# 071/16) and was performed following the U.S. National Institutes of Health Guide for the Care and Use of Laboratory Animals. C57BL/6J background homozygous LDLR KO mice were purchased from Jackson Laboratory. Mice were housed in a conventional animal facility at 22 °C ± 2 deg. C under a 12-h light/dark cycle with free access to commercial chow (Nuvilab CR1) and drinking water. Plasma lipids (TG, TC) were enzymatically determined (Labtest Brazil) in the basal period after collecting 12-h fasting blood samples from the tail vein in heparinized capillary tubes. Twelve-week-old male LDLR KO mice were randomly divided into two groups fed *ad libitum*, during 90 days, pelleted chow containing either 0.06% (low-sodium; LS) or 0.5% normal sodium chloride (NS) (Teklad—Envigo). The amount of sodium chloride in the LS was sufficient for normal mice growth rate. Diets were composed of the following nutrients (g/100 g): casein (28.7), sucrose (31.3), corn starch (20.0), soybean oil (6.0), cellulose (9.79), Vitamin mix (Teklad—1.0), and ethoxyquin (1, 5). Body weight and food ingestion were monitored weekly.

Body weight, hematocrit, 24 h-urinary sodium (UNa) excretion, BP, plasma TC, TG, and insulin tolerance test (kITT) were determined at the end of the experimental protocol. UNa obtained in individual metabolic cages with water and food *ad libitum* was determined in FC 280 flame spectrophotometer (CELM). Systolic BP was assessed in conscious animals utilizing a standard tail-cuff technique and performed by photoplethysmography (BP-2000-M2 Blood Pressure Analysis System—Visitech Systems). Resting animals were preconditioned during 5 to 7 days to the method before final analyses. Eight consecutive measurements were recorded in order to obtain mean values.

### Insulin tolerance test (kITT)

The kITT was performed at the end of the 10th week. Briefly, after 4 h of food deprivation, tail blood samples were obtained (0 min—basal glucose concentration) and at 10, 20, and 30 min after an intraperitoneal injection of regular human insulin (1 UI/kg of body mass; Eli Lilly and company). Glucose concentration was measured by Accu Check glucometer (Accu Check Performa—Roche). The constant rate of blood glucose disappearance (kITT) was determined by linear regression.

### Fast protein liquid chromatography (FPLC)

Lipoprotein profile was determined by FPLC utilizing 100 μl of animal plasma on a NS or LS in Superose 6HR 10/30 column (FPLC System, Pharmacia), with elution in constant flow of 0.5 ml/min in Tris buffer (Tris 10 mM, NaCl 150 mM, EDTA 1 mM e NaN3 0.03% - pH 7,4). TG and TC content were enzymatically (Labtest) determined in lipoprotein fractions.

### Isolation of the gastrocnemius muscle

After euthanasia (sodium thiopental; 150 mg/kg of body weight, i.p.), left and right gastrocnemius were harvested from each mouse, dissected, and stored at −80 °C.

### RT-qPCR

Total RNA was extracted from left gastrocnemius by Trizol reagent (Invitrogen Life Technologies, Carlsbad, CA, USA) and purified by RNeasy Mini Kit (Qiagen, USA). Total RNA (2 μg) was reverse transcribed to cDNA using High Capacity RNA-to-cDNA kit (Applied Biosystems) according to the manufacturer's instructions. Real-time quantitative PCR was performed by Taqman assays (Applied Biosystems). The following TaqMan Gene Expression Assays were used: *Fabp3* (Fatty acid binding protein 3, skeletal and cardiac muscles, Mm02342495_m1), *Cd36* (CD36 antigen, Mm00432403_m1), *Cpt1b* (Carnitine palmitoyltransferase 1b, muscle, Mm00487191_g1), *Acacb* (Acetyl-Coenzyme A carboxylase beta, Mm01204671_m1), Irs1 (Insulin receptor substrate 1, Mm01278327_m1), *Akt1* (Thymoma viral proto-oncogene 1, Mm01331626_m1), *Slc2a4* (Solute carrier family 2 facilitated glucose transporter, member 4, Mm00436615_m1), *Lpl* (Lipoprotein lipase, Mm00434764_m1), *Ppargc1a* (Peroxisome proliferative activated receptor, gamma, coactivator 1 alpha, Mm01208835_m1), *Prkaa1* (Protein kinase AMP-activated catalytic subunit alpha 1, Mm01296700_m1) in the StepOne Plus—Real-Time PCR System (Applied Biosystems by Life Technologies). The relative expression of each gene was normalized to the housekeeping gene and *B2m* (Beta-2 microglobulin, Mm00437762_m1) and relative quantification analysis was performed with StepOne Software 2.0 (Applied Biosystems) using the comparative cycle threshold (Ct) (2^−ΔΔCt^) method (50).

### Western blotting

Protein content of AKT, pAKT, and GLUT4 in the right gastrocnemius was determined by western blot. Briefly, 50 μg of total muscle homogenate was submitted to electrophoresis into a 10% polyacrylamide gel and after transference to a nitrocellulose membrane, immunoblot was performed by using total and pAKT Ab (1:1000 - Sc-8312 and Sc-7985-R, Santa Cruz Biotechnology) and GLUT4 Ab (1:3000 - 07-1404, Milipore Corporation). After incubation with secondary anti-rabbit Ab conjugated to peroxidase (1:5000), membranes were incubated with chemiluminescence reaction enhancer (ECL). The Ponceau staining was utilized to normalize the optical density of each band. Results were presented in arbitrary units corrected per gram of tissue.

### Lipidomics

Lipids were extracted from mice gastrocnemius as previously described by Yoshida *et al.* (2008). Five-hundred microliters of total gastrocnemius homogenate (100 mg/1 ml) was added to 500 μl of methanol and butylated hydroxytoluene (0.1 mM), 1.5 ml of chloroform; ethyl acetate (4:1 ratio) and the following internal standards of lipids species: ceramide (Cer) d18:1/17:0, sphingomyelin (SM) d18:1/17:0, cardiolipin (CL) 14:0/14:0/14:0/14:0, phosphatidylcholine (PC) 17:0/17:0, phosphatidylethanolamine (PE) 17:0/17:0, phosphatidylserine (PS) 17:0/17:0, phosphatidylglycerol (PG) 17:0/17:0, phosphatidic acid (PA) 17:0/17:0, LysoPC (LPC) 17:0, Lyso PE 17:1, TG 17:0/17:0/17:0 from Avanti Polar Lipids and cholesteryl ester (CE) 10:0 from Sigma Aldrich. The mixture was vortexed and centrifugated (1500*g*/2 min) at 4 °C and the organic phase was removed and dried under nitrogen flow. Total lipid extracts were dried under nitrogen and eluted in isopropanol (100 μl). For global lipidomics analysis, total gastrocnemius lipid extract was analyzed by ultrahigh-performance liquid chromatography (UHPLC, Nexera, Shimadzu, Kyoto, Japan) coupled to ESI-Q-TOF-MS (TripleTOF 6600, Sciex, Concord, Estados Unidos) as previously described by Chaves-Filho *et al.* (2019) (51). One microliter from this volume was injected into a CORTECS C18 column (1.6 μm, 2.1 mm i.d. × 100 mm) with a 0.2 ml min^−1^ of flow rate and the oven temperature at 35 °C. For reverse-phase chromatography, the mobile phase A consisted of water/acetonitrile (60:40 ratio) and mobile phase B of isopropanol/acetonitrile/water (88:10:2). Ammonium acetate or ammonium formate at a final concentration of 10 mM was used in mobile phase A and B for experiments in both negative and positive ionization modes. The separation of lipids was achieved in linear gradient during 20 min, the first 10 min from 40% to 100% B, maintained at 100% from 10 to 12 min, decreased from 100% to 40% from 12 to 13 min, and maintained at 40% B from 13 to 20 min. Mass spectrometry experiments were conducted in positive and negative modes with a scan range of the mass-to-charge ratio of 200 to 2000 Da. Data acquisition was performed by using Analyst 1.7.1 with a cycle time of 1.05 s with 100 ms acquisition time for MS1 scan and 25 ms acquisition time to obtain the top 36 precursor ions. Ion spray voltage of −4.5 kV and 5.5 kV, for negative and positive modes, respectively, and the cone voltage at ±80 V were set to analysis. Additional parameters included curtain gas set at 25 psi, nebulizer and heater gases at 45 psi, and interface heater of 450 °C. PeakView software was used for the identification of lipid molecular species in Information-Dependent Acquisition (IDA). Lipid molecular species were identified according to their exact mass, specific fragments, and neutral losses (16 chaves). This process was performed manually using an in-house manufactured Excel-based macro. The area of lipid species was obtained by MS data using MultiQuant. Lipids species concentrations were calculated by multiplying the area ratio of lipid species by the concentration of their corresponding internal standard. For lipid classes lacking internal standards such as DG, FFA, and PI, external calibration curves relative to PC (17:0/17:0) were performed with DG (17:0/17:0), FFA (17:0), and PI (14:1/17:0). Acylcarnitines (AC) were quantified relative to LPC (17:0) without an external calibration, and therefore, the data is qualitative and values can only be compared between samples but not with other lipids.

## Data availability

All data reported is included in the article and in the supplementary information section. Upon personal request to the authors, all raw data can be kindly shared. This article includes original data and has not been published elsewhere.

## Conflict of interest

The authors declare that they have no conflicts of interest with the contents of this article.
