# Supplementary material for: Dietary sodium restriction alters muscle lipidomics that relates to insulin resistance in mice
Source: J Biol Chem. 2021 Jan 29;296:100344. doi: 10.1016/j.jbc.2021.100344 (PMC7949138; doi:10.1016/j.jbc.2021.100344)
Supplement: Supporting Information [file mmc1.pdf]

Supporting Information

Table 1. Phosphatidylethanolamine (PE) species identified in the gastrocnemius of LDLR KO male mice fed either the normal sodium (NS; n = 7) or the low-sodium diet (LS; n = 7). Part 1.

	**NS**			**LS**			***P***
**PE (16:0/18:1)**	0.2660	±	0.043	0.3312	±	0.021	0.160
**PE (16:0/18:2)**	0.5750	±	0.161	0.5337	±	0.033	0.898
**PE (16:0/20:3)**	0.1177	±	0.030	0.0769	±	0.004	0.443
**PE (16:0/22:4)**	0.9157	±	0.220	0.7426	±	0.025	0.250
**PE (16:0/20:4)**	1.0722	±	0.283	1.0567	±	0.060	0.701
**PE (16:0/22:6)**	9.9811	±	2.000	10.5588	±	0.412	0.371
**PE (16:0/22:5)**	1.4665	±	0.334	1.4272	±	0.058	0.443
**PE (16:1/18:0)**	0.2301	±	0.061	0.1655	±	0.010	**0.021**
**PE (16:1/22:6)**	0.5409	±	0.089	0.5190	±	0.044	1.000
**PE (17:0/22:6)**	0.3807	±	0.076	0.3498	±	0.038	0.798
**PE (18:0/18:1)**	0.4524	±	0.567	0.4462	±	0.484	0.898
**PE (18:0/18:2)**	1.1170	±	0.302	1.0181	±	0.063	0.898
**PE (18:0/20:3)**	0.1588	±	0.040	0.1582	±	0.012	0.898
**PE (18:0/22:4)**	0.4470	±	0.112	0.4340	±	0.019	0.701
**PE (18:0/20:4)**	2.9129	±	0.728	2.8888	±	0.218	0.798
**PE (18:0/22:6)**	18.6180	±	3.420	20.3365	±	1.290	1.000
**PE (18:0/22:5)**	3.1309	±	0.580	3.2288	±	0.131	0.798
**PE (18:1/18:1)**	0.2449	±	0.067	0.3274	±	0.015	0.250
**PE (18:1/18:2)**	0.3795	±	0.132	0.3642	±	0.027	1.000
**PE (18:1/24:0)**	0.8638	±	0.020	0.0755	±	0.007	0.701
**PE (18:1/20:4)**	1.6324	±	0.359	1.7087	±	0.072	0.609
**PE (18:1/22:6)**	5.6297	±	1.250	5.5887	±	0.281	0.798
**PE (18:1/22:5)**	0.5764	±	0.092	0.4502	±	0.033	0.201
**PE (18:2/18:2)**	0.1487	±	0.050	0.1426	±	0.007	0.798
**PE (18:2/20:4)**	3.3875	±	0.554	2.8658	±	0.407	0.898
**PE (18:2/22:6)**	2.0812	±	0.438	1.9399	±	0.102	1.000
**PE (18:2/22:5)**	0.0867	±	0.019	0.0919	±	0.005	0.443
**PE (19:0/22:6)**	0.1205	±	0.020	0.1534	±	0.010	0.443
**PE (20:1/22:6)**	0.1607	±	0.036	0.1731	±	0.011	1.000
**PE (22:4/22:6)**	1.1434	±	0.221	0.8747	±	0.072	0.125
**PE (20:4/22:6)**	0.19551	±	0.026	0.1416	±	0.010	0.201
**PE (22:5/22:6)**	0.1532	±	0.021	0.1475	±	0.016	0.798

Table 2. Plasmenil Phosphatidylethanolamine (PE) species identified in the gastrocnemius of LDLR KO male mice fed either the normal sodium (NS; n = 7) or the low-sodium diet (LS; n = 7).

	NS			LS			*P*
**pPE (p16:0/18:1)**	0.4826	±	0.066	0.5527	±	0.045	0.160
**pPE (p16:0/22:4)**	0.3978	±	0.077	0.3537	±	0.013	0.523
**pPE (p16:0/20:4)**	0.7108	±	0.178	0.7169	±	0.036	0.898
**pPE (p16:0/22:6)**	10.5008	±	2.560	10.2824	±	0.834	1.000
**pPE (p16:0/22:5)**	1.5650	±	0.377	1.6925	±	0.113	0.609
**pPE (p17:0/18:1)**	0.0903	±	0.023	0.1083	±	0.009	0.523
**pPE (p18:0/18:1)**	0.4128	±	0.477	2.8373	±	0.264	**0.011**
**pPE (p18:0/22:4)**	0.4487	±	0.128	0.3978	±	0.022	0.443
**pPE (p18:0/20:4)**	0.6119	±	0.142	0.5925	±	0.0336	1.000
**pPE (p18:0/22:6)**	3.5425	±	0.830	3.8973	±	0.223	0.443
**pPE (p18:0/22:5)**	0.6468	±	0.153	0.7967	±	0.039	0.371
**pPE (p18:0:1/16:0)**	0.6299	±	0.159	0.6005	±	0.045	0.250
**pPE (p18:0:1/18:1)**	3.2096	±	0.653	3.3515	±	0.299	0.701
**pPE (p18:0:1/18:2)**	0.2228	±	0.052	0.2395	±	0.015	0.523
**pPE (p18:0:1/20:4)**	0.0473	±	0.014	0.0559	±	0.012	0.523
**pPE (p18:0:1/22:6)**	2.6674	±	0.574	2.6973	±	0.201	1.000
**pPE (p18:0:1/22:5)**	0.2652	±	0.055	0.2857	±	0.020	0.701
**pPE (p18:0:2/18:1)**	0.3060	±	0.090	0.3371	±	0.029	0.609
**pPE (p18:0:2/22:6)**	0.4162	±	0.157	0.4589	±	0.046	1.000
**pPE (p20:0/22:6)**	0.1266	±	0.041	0.1213	±	0.009	0.898

Table 3. Phosphatidylglicerine (PG) species identified in the gastrocnemius of LDLR KO male mice fed either the normal sodium (NS; n = 7) or the low-sodium diet (LS; n = 7).

	NS			LS			*P*
**PG (16:0/18:1)**	0,2541	±	0.063	0,3719	±	0.018	0.307
**PG (16:0/18:2)**	0.0440	±	0.012	0.0571	±	0.002	0.609
**PG (16:0/20:4)**	0.0156	±	0.003	0.0159	±	0.002	0.609
**PG (16:1/18:1)**	0.0051	±	0.000	0.0067	±	0.0004	0.097
**PG (18:0/18:1)**	0.3313	±	0.066	0.3662	±	0.021	0.701
**PG (18:0/18:2)**	0.2119	±	0.057	0.2283	±	0.081	1.000
**PG (18:1/18:2)**	0.0499	±	0.014	0.0401	±	0.004	0.898

Table 4. Sphingomyelin (SM) species identified in the gastrocnemius of LDLR KO male mice fed either the normal sodium (NS; n = 7) or the low-sodium diet (LSD; n = 7).

	NS			LS			*P*
**SM (d18:0/16:0)**	0.0349	±	0.003	0.0325	±	0.004	0.701
**SM (d18:1/16:0)**	0.3646	±	0.047	0.3721	±	0.044	0.443
**SM (d18:1/18:0)**	1.5587	±	0.190	1.7683	±	0.136	0.443
**SM (d18:1/20:0)**	0.2046	±	0.035	0.2388	±	0.026	0.701
**SM (d18:1/22:0)**	0.5580	±	0.115	0.6716	±	0.062	0.798
**SM (d18:1/22:1)**	0.0609	±	0.010	0.0642	±	0.007	0.523
**SM (d18:1/23:0)**	0.1566	±	0.021	0.1609	±	0.017	0.609
**SM (d18:1/24:0)**	0.6884	±	0.132	0.7956	±	0.087	0.701
**SM (d18:1/24:1)**	1.3333	±	0.265	1.3824	±	0.161	0.898
**SM (d18:1/24:2)**	0.1794	±	0.034	0.1872	±	0.027	0.701

Table 5. Ceramide (Cer) species identified in the gastrocnemius of LDLR KO male mice fed either the normal sodium (NS; n = 7) or the low-sodium diet (LS; n = 7). Part 1.

	NS			LS			***P***
**1G-Cer (d18:0/20:1-OH)**	0.0300	±	0.004	0.0325	±	0.003	0.523
**1G-Cer (d18:0/22:0-OH)**	0.0303	±	0.0004	0.0334	±	0.0002	0.307
**1G-Cer (d18:0/22:1-OH)**	0.1615	±	0.027	0.1579	±	0.022	0.701
**1G-Cer (d18:0/23:1-OH)**	0.0112	±	0.001	0.0097	±	0.001	0.798
**1G-Cer (d18:0/24:0-OH)**	0.0111	±	0.001	0.0130	±	0.001	0.371
**1G-Cer (d18:0/24:1-OH)**	0.2865	±	0.043	0.2924	±	0.036	0.609
**1G-Cer (d18:0/24:2-OH)**	0.0215	±	0.003	0.0195	±	0.002	0.609
**1G-Cer (d18:1/22:0-OH)**	0.0014	±	0.0004	0.0011	±	0.0002	0.307
**1G-Cer (d18:1/23:0-OH)**	0.0031	±	0.0006	0.0034	±	0.0005	0.609
**1G-Cer (d18:0/24:0)**	0.0105	±	0.002	0.0110	±	0.001	0.701
**1G-Cer (d18:1/20:0)**	0.0101	±	0.001	0.0100	±	0.001	0.701
**1G-Cer (d18:1/22:0)**	0.0359	±	0.005	0.0328	±	0.005	0.523
**1G-Cer (d18:1/23:0)**	0.0145	±	0.002	0.0119	±	0.002	0.523
**1G-Cer (d18:1/24:0)**	0.2028	±	0.032	0.1751	±	0.030	0.701
**1G-Cer (d18:1/24:1)**	0.1070	±	0.017	0.0970	±	0.016	0.523
**1G-Cer (d18:1/24:2)**	0.0103	±	0.0017	0.0092	±	0.001	0.798
**Sulfatide Cer (d18:1/18:0)**	0.0036	±	0.0005	0.0038	±	0.0005	0.125
**Sulfatide Cer (d18:1/20:0)**	0.0021	±	0.0003	0.0028	±	0.0005	0.097
**Sulfatide Cer (d18:1/22:0)**	0.0060	±	0.001	0.0060	±	0.0008	0.701
**Sulfatide Cer (d18:1/23:0)**	0.0019	±	0.0004	0.0021	±	0.0002	0.443

Table 5. Ceramide (Cer) species identified in the gastrocnemius of LDLR KO male mice fed either the normal sodium (NS; n = 7) or the low-sodium diet (LS; n = 7). Part 2.

	NS			LS			***P***
**Sulfatide Cer (d18:1/24:0)**	0.0355	±	0.005	0.0311	±	0.005	0.701
**Sulfatide Cer (d18:1/24:1)**	0.0361	±	0.007	0.0321	±	0.005	0.798
**Sulfatide Cer (d18:2/24:1)**	0.0042	±	0.001	0.0046	±	0.0008	0.307
**Sulfatide Cer (d18:1/22:0-OH)**	0.0030	±	0.0008	0.0045	±	0.0005	0.201
**Sulfatide Cer (d18:1/24:0-OH)**	0.0050	±	0.001	0.0063	±	0.0006	0.250
**Cer (d18:1/18:0)**	0.2088	±	0.040	0.2663	±	0.015	0.443
**Cer (d18:1/22:0)**	0.0088	±	0.001	0.0112	±	0.0007	0.250
**Cer (d18:1/24:0)**	0.0093	±	0.002	0.0119	±	0.001	0.160
**Cer (d18:1/24:1)**	0.0231	±	0.004	0.02652	±	0.002	0.609
**Cer (d18:1/24:2)**	0.0043	±	0.001	0.00499	±	0.0006	0.201

Table 6. Diacylglycerol (DG) species identified in the gastrocnemius of LDLR KO male mice fed either the normal sodium (NS; n = 7) or the low-sodium diet (LS; n = 7)

	NS			LS			***P***
**DG (16:1/18:1)**	2.6338	±	0.339	3.6384	±	0.366	0.250
**DG (18:2/18:2)**	0.9752	±	0.259	1.1888	±	0.216	0.609
**DG (18:1/18:2)**	3.4143	±	0.524	4.4584	±	0.490	0.371
**DG (18:1/18:1)**	1.8148	±	0.223	1.9748	±	0.207	0.701

Table 7. Triglycerides (TG) species identified in the gastrocnemius of LDLR KO male mice fed either the normal sodium (NS; n = 7) or the low-sodium diet (LS; n = 7). Part 1.

	NS			LS			***P***
**TG (12:0/16:1/18:2)**	0,0687	±	0.025	0,0759	±	0.013	1.000
**TG (14:0/16:0/16:1)**	0,3146	±	0.220	0,3466	±	0.044	0.798
**TG (14:0/16:0/18:1)**	0,2417	±	0.319	0,5257	±	0.122	0.898
**TG (14:0/16:1/18:1)**	0,3945	±	0.221	0,4286	±	0.066	0.609
**TG (14:0/16:1/16:1)**	0,6152	±	0.264	0,7356	±	0.085	0.443
**TG (14:0/18:1/18:1)**	0,5218	±	0.751	1.6342	±	0.382	0.898
**TG (14:0/18:2/18:2)**	0.6987	±	0.212	0.9470	±	0.130	0.943
**TG (14:1/16:0/16:1)**	0.0147	±	0.031	0.0678	±	0.016	1.000
**TG (14:1/16:1/18:1)**	0.3803	±	0.166	0.5099	±	0.060	0.371
**TG (14:1/16:1/18:2)**	0.5051	±	0.133	0.6282	±	0.075	0.609
**TG (14:1/16:1/16:1)**	0.2456	±	0.079	0.2996	±	0.043	0.523
**TG (14:1/18:2/18:2)**	0.6413	±	0.144	0.8332	±	0.105	0.307
**TG (15:0/16:1/18:1)**	0.0565	±	0.018	0.0534	±	0.006	0.898
**TG (15:0/16:1/18:2)**	0.1142	±	0.041	0.1352	±	0.013	0.898
**TG (15:0/18:1/18:2)**	0.2680	±	0.103	0.3604	±	0.043	0.701
**TG (15:0/18:1/18:1)**	0.0341	±	0.052	0.1123	±	0.024	0.701
**TG (15:0/18:2/18:2)**	0.0312	±	0.006	0.0291	±	0.005	1.000
**TG (16:0/16:1/17:1)**	0.0183	±	0.075	0.1935	±	0.022	0.701
**TG (16:0/16:1/18:1)**	4.3697	±	2.120	6.6899	±	0.833	0.609
**TG (16:0/16:1/22:6)**	0.1552	±	0.046	0.2200	±	0.029	0.701
**TG (16:0/16:1/16:1)**	1.6426	±	0.748	2.2424	±	0.247	0.523
**TG (16:0/17:1/18:1)**	0.2712	±	0.113	0.3871	±	0.044	0.701
**TG (16:0/17:1/18:2)**	0.1310	±	0.033	0.1176	±	0.013	0.898
**TG (16:0/18:0/18:1)**	0.4230	±	0.390	0.5933	±	0.082	0.898
**TG (16:0/18:0/18:2)**	0.7326	±	1.010	2.1276	±	0.531	0.701
**TG (16:0/18:1/18:2)**	6.1253	±	2.700	9.2644	±	1.170	0.609
**TG (16:0/18:1/19:1)**	0.0517	±	0.060	0.1091	±	0.022	0.701

Table 7. Triglycerides (TG) species identified in the gastrocnemius of LDLR KO male mice fed either the normal sodium (NS; n = 7) or the low-sodium diet (LS; n = 7). Part 2.

	NS			LS			***P***
**TG (16:0/18:1/20:1)**	0.4536	±	0.460	0.8374	±	0.238	0.798
**TG (16:0/18:1/22:6)**	0.1398	±	0.046	0.211	±	0.031	0.443
**TG (16:0/18:1/18:1)**	5.3333	±	1.730	7.4239	±	0.926	1.000
**TG (16:0/18:2/22:4)**	0.0415	±	0.040	0.0929	±	0.025	0.609
**TG (16:0/18:2/22:6)**	0.1733	±	0.040	0.2911	±	0.025	0.371
**TG (16:0/18:2/18:2)**	0.3569	±	0.474	1.0290	±	0.303	0.701
**TG (16:0/16:0/16:1)**	1.0012	±	0.756	1.1728	±	0.171	0.798
**TG (16:0/16:0/18:1)**	2.0052	±	1.670	2.9952	±	0.400	1.000
**TG (16:0/16:0/16:0)**	0.1914	±	0.107	0.3591	±	0.048	0.371
**TG (16:1/17:1/18:2)**	0.2188	±	0.070	0.3100	±	0.033	0.898
**TG (16:1/18:1/18:2)**	8.6219	±	2.700	12.0870	±	1.170	0.609
**TG (16:1/18:1/18:1)**	2.7634	±	0.778	3.0303	±	0.393	0.609
**TG (16:1/18:2/18:3)**	0.6249	±	0.173	0.9371	±	0.139	0.307
**TG (16:1/18:2/22:5)**	0.0119	±	0.010	0.0350	±	0.010	0.443
**TG (16:1/18:2/18:2)**	5.9665	±	1270	6.6006	±	0.853	0.523
**TG (16:1/16:1/17:1)**	0.0622	±	0.017	0,0853	±	0.011	0.798
**TG (16:1/16:1/18:1)**	6.8728	±	1.830	8.5875	±	0.829	0.701
**TG (16:1/16:1/18:2)**	4.1106	±	0.840	4.6548	±	0.515	0.609
**TG (16:1/16:1/18:3)**	0.1380	±	0.036	0.1578	±	0.029	0.609
**TG (16:1/16:1/20:4)**	0.5831	±	0.102	0.5774	±	0.081	0.201
**TG (16:1/16:1/16:1)**	1.9518	±	0.507	2.4398	±	0.283	0.609
**TG (17:0/18:1/18:2)**	0.0364	±	0.044	0.0955	±	0.021	0.701
**TG (17:1/18:1/18:2)**	0.2894	±	0.094	0.3623	±	0.042	0.898
**TG (17:1/18:1/18:1)**	0.3099	±	0.104	0.3808	±	0.043	0.701
**TG (17:1/18:2/18:2)**	0.1250	±	0.038	0.1698	±	0.019	0.898
**TG (18:0/18:1/18:2)**	0.0446	±	0.011	0.0448	±	0.007	1.000
**TG (18:0/18:1/18:1)**	0.7749	±	0.309	0.9041	±	0.114	0.898
**TG (18:1/18:2/18:3)**	0.8532	±	0.199	1.1533	±	0.125	0.250
**TG (18:1/18:2/19:1)**	0.0889	±	0.034	0.0958	±	0.012	0.898
**TG (18:1/18:2/20:1)**	0.2874	±	0.137	0.3563	±	0.052	0.798
**TG (18:1/18:2/20:2)**	0.1743	±	0.066	0.2608	±	0.035	0.443
**TG (18:1/18:2/20:3)**	0.1834	±	0.074	0.2137	±	0.036	0.798
**TG (18:1/18:2/22:4)**	0.0979	±	0.037	0.1202	±	0.021	0.701
**TG (18:1/18:2/20:4)**	0.4301	±	0.127	0.5883	±	0.076	0.701
